# Society Meetings

**Published:** 1903-08

**Authors:** 


### SOCIETY MEETINGS.

The Medical Association of Central New York will hold its 36th annual meeting at Auburn, September 22, 1903, under the presidency of Dr. John L. Heffron, of Syracuse. The president asks that titles of papers be sent to him at once, as the program will be issued early in September.

---

The Lake Keuka Medical and Surgical Association will hold its annual meeting at Grove Springs, Lake Keuka, August 11 and 12, 1903. Dr. Arthur W. Booth, of Elmira, is the secretary, to whom titles of papers should be sent.

---

The Lake Erie Medical Society, held its regular quarterly meeting at Angola, N. Y., July 3, 1903. The following program was observed: Urine and its analysis, J. S. Wright; a paper by Dr. Moore, Dunkirk; Senile prostate, B. H. Daggett, Buffalo; a paper by Matthew D. Mann, Buffalo.

